# The bidirectional relationship between activities of daily living and frailty during short-and long-term follow-up period among the middle-aged and older population: findings from the Chinese nationwide cohort study

**DOI:** 10.3389/fpubh.2024.1382384

**Published:** 2024-04-30

**Authors:** Xiaoping Li, Xiaoguang Li, Lu Sun, Liu Yang, Congzhi Wang, Ting Yuan, Yunxiao Lei, Jing Li, Mingming Liu, Dongmei Zhang, Ying Hua, Haiyang Liu, Lin Zhang

**Affiliations:** ^1^Department of Emergency and Critical Care Nursing, School of Nursing, Wannan Medical College, Wuhu, China; ^2^National Center For Occupational Safety and Health, National Health Commission of the People’s Republic of China, Beijing, China; ^3^Department of Internal Medicine Nursing, School of Nursing, Wannan Medical College, Wuhu, China; ^4^Obstetrics and Gynecology Nursing, School of Nursing, Wannan Medical College, Wuhu, China; ^5^Department of Surgical Nursing, School of Nursing, Wannan Medical College, Wuhu, China; ^6^Department of Pediatric Nursing, School of Nursing, Wannan Medical College, Wuhu, China; ^7^Rehabilitation Nursing, School of Nursing, Wanna Medical College, Wuhu, China; ^8^Student Health Center, Wannan Medical College, Wuhu, China

**Keywords:** frailty, activities of daily living, bidirectional relationship, middle-aged and older adults, basic activities of daily living, instrumental activities of daily living

## Abstract

**Objective:**

Frailty and activities of daily living (ADL) disability are common conditions among older population. Studies on the bidirectional relationship between frailty and ADL are limited. The current study examined the cross-sectional and longitudinal associations between frailty and ADL in middle-aged and older Chinese individuals.

**Methods:**

The data was collected through the China Health and Retirement Longitudinal Study (CHARLS), conducted in 2011, 2013, and 2015, encompassing 17,284 individuals aged ≥45 years. We excluded individuals without follow-up data. 2,631 participants finished the baseline survey. The definition of ADL disability encompasses difficulty in engaging in either basic activities of daily living (BADL) or instrumental activities of daily living (IADL). Frailty was assessed according to the Fried criteria. Logistic regression was utilized to examine odds ratios (ORs) and 95% confidence intervals (CIs) for assessing the cross-sectional relationships between ADL with frailty at baseline. The prediction effects were explored using Cox proportional hazards analysis, testing hazard ratios (HRs) and 95%CIs.

**Results:**

In cross-sectional analysis, BADL [OR = 6.660 (4.519–9.815)], IADL [OR = 5.950 (4.490–7.866)], and ADL [OR = 5.658 (4.278–7.483)] exhibited significant associations with frailty; frailty demonstrated significant associations with BADL [OR = 6.741 (4.574–9.933)], IADL [OR = 6.042 (4.555–8.016)] and ADL [OR = 5.735 (4.333–7.591)]. In longitudinal analysis, IADL and ADL were significantly associated with frailty in participants without baseline frailty in the short-term period [IADL: HR = 1.971 (1.150–3.379), ADL: HR = 1.920 (1.146–3.215)], IADL exhibited a significant association with frailty in the long-term period [HR = 2.056 (1.085–3.895)]. There was no significant link observed between frailty and an elevated risk of disability onset in BADL, IADL and ADL during the short-term period. When considering the long-term perspective, frailty exhibited a significant association with an elevated risk of disability onset in BADL [HR= 1.820 (1.126–2.939)] and IADL [HR = 1.724 (1.103–2.694)].

**Conclusion:**

In middle-aged and older adults, ADL and IADL disability predicted frailty after 2-year follow-up, IADL disability predicted frailty after 4-year follow-up. Moreover, frailty did not predict BADL, IADL and ADL disability after 2-year follow-up. However, frailty predicted BADL and IADL disability after 4-year follow-up.

## Introduction

Frailty is a geriatric syndrome with multiple dimensions, characterized by an increased vulnerability to different stressors. Frailty is associated with poor health outcomes and signifies a decline in physiological reserve and function, which becomes increasingly prevalent with advancing age (1, 2). Frailty is prevalent among middle-aged and older people. Previous studies have reported varying prevalence rates of frailty in middle-aged and older population of 3% in United Kingdom (3), 4.1% in Europe (4), 11.8% in Chilean and 3.1% in China (5). Older population has a higher prevalence of frailty. According to a comprehensive meta-analysis (included studies of cross-sectional and cohort), the prevalence of frailty among Chinese older community-dwelling population was 7–20% (2, 6–8), 11% for females and 8% for males (6). The presence of frailty is associated with elevated rates of hospitalization, dementia, disability, and early mortality (9–11), and presents significant challenges to global healthcare systems. With the aging of China’s population, frailty has emerged as one pressing concern for healthcare systems, posing a significant public health burden. Relevant literature have extensively documented a large number of risk factors for frailty, such as advanced ages, unmarried, women, rural areas, living alone, lower levels of education, worse economic conditions, three or more chronic diseases, and ADL disability (2, 6, 7, 12).

According to the explanations provided in medical and rehabilitation literature, the Activities of Daily Living (ADL) refers to activities focused on self-care and personal hygiene (13). ADL are widely acknowledged as reliable indicators of functional restrictions among older individuals and are commonly categorized into basic activities of daily living (BADL) and instrumental activities of daily living (IADL) (14). BADL reflects the fundamental self-care ability that individual must consistently perform repeatedly every day to maintain their independence, while IADL refers to the more complex ability to participate in social activities and live independently (15). The adverse outcomes of ADL disability encompass hospitalization (16), mortality (17), and diminished levels of quality of life (18), among others. Several factors were associated with ADL disability, including age, chronic pain, polypharmacy, being separated/divorced, physical activity, and frailty (19–21). Most of the studies encompassed individuals aged ≥65 years, 26% of community-dwelling older people in China exhibited limitations in ADL (17), 19.02% limitations in BADL and 25.29% limitations in IADL (22). The prevalence rate of BADL disability among middle-aged and older community-dwelling adults in China was found to be 16.7%, IADL disability was 21.5% (23), and ADL disability was 19.4% (24). 2006–2018 study in United States including middle-aged and older participants showed that 25.4% presented IADL impairments, and the prevalence of impairments in IADL was highest among females and older individuals (25). A cross-sectional study of older adults residing in Brazilian communities in 2015–2016 found that the prevalence of IADL and BADL disabilities was 45.1 and 13.5%, respectively (26). A study conducted in India population aged ≥60 years revealed the prevalence of ADL and IADL were 19.89 and 45.00%, respectively (27). Other studies have described a prevalence rate of 48.5% for ADL disability among middle-aged and older individuals with chronic obstructive pulmonary disease (COPD) (28), the prevalence of IADL and BADL disability among cancer patients was reported as 54.6 and 36.7%, respectively (29).

Previous studies have researched the association of ADL and frailty. A meta-analysis performed for 31 prospective studies (9) showed a prospective association between frailty and ADL disability, with frailty leading to a significantly higher risk of BADL disability (OR: 2.05, 95% CI: 1.73–2.44) and IADL disability (OR: 2.52, 95% CI: 2.08–3.06); frailty increases the risk of losing ADL by 1.6 to 2.0 times. Another meta-analysis also showed that associations persisted among community-dwelling middle-aged and older population (30). Meta-analysis encompassed 20 studies that examined the risks of disability in IADL and ADL, it was found that frail older individuals were more prone to developing or exacerbating disability in IADL and ADL, the pooled OR and pooled HR exhibited statistically significant results (31). The cross-sectional study reported that frailty group exhibited significantly worse scores for the ADL than the non-frailty group in community-dwelling older adults (*p* < 0.05) (32). Analysis of Italian population cohort, the incident risk of IADL disability was increased in frailty individuals (HR: 2.56, 95%CI: 1.58–4.16) and ADL (HR: 3.58, 95%CI: 1.97–6.52) after a four-year follow-up; pre-frailty participants displayed a higher incidence of frailty compared to those without frailty at follow-up entry (33). On the other side, a meta-analysis comprising 14 studies (encompassing cross-sectional studies and cohort studies) (6) reported that having ADL disability was a risk factor for frailty among the older Chinese population in communities. Another meta-analysis collected cross-sectional data on the frailty prevalence among rural older individuals and revealed that ADL disability was significantly associated with frailty (34).

However, the existing studies mostly focus on the prevalence and risk factors of frailty (2, 6, 7, 34), whereas the effect of ADL on the incidence of frailty in longitudinal studies has been poorly understood (1). In addition, previous research primarily examined the unidirectional associations between ADL and frailty, and were limited by cross-sectional designs or small sample sizes. Considering the shared risk factors and pathophysiological mechanisms involved, our study hypothesizes a bidirectional relationship between frailty and ADL. It is imperative to prospectively investigate the bidirectional association of frailty and ADL disability utilizing a large and representative sample, to provide new references for the development of frailty and ADL disability interventions.

In the study, we utilized a four-year (2011 to 2015) longitudinal dataset from a nationally representative sample of community-dwelling adults in China, aged middle-aged and older. We aimed to explore the bidirectional relationship of ADL and frailty over short-term (2-year, 2011–2013) and long-term (4-year, 2011–2015) periods. We hypothesized that baseline ADL disability is predictive of the following frailty occurrence. Similarly, baseline frailty is predictive of subsequent changes in ADL disability.

## Method

### Participants

The research is based on the China Health and Retirement Longitudinal Study (CHARLS), which collected data from a cohort of 17,284 individuals aged ≥45 years in 2011 (Wave 1). Subsequent data collection occurred in 2013 and 2015 (Waves2 and Waves3). Participants were selected for in-person interviews, computer-aided personal interview (CAPI), and structured questionnaires conducted biennially. This study utilized longitudinal data from individuals who participated in Wave1, Wave2, and Wave3. The following exclusion criteria were formulated for this study: (1) lack of components of frailty data, (2) lack of components of activities of daily living disability data, and (3) lack of sex/age/current residence/educational level/marital status/alcohol drinking/smoking status/taking activities/physical exercise/BMI/chronic diseases data. Furthermore, participants without follow-up data were excluded. A total of 2,631 participants finished the baseline assessments, with 1936 participants finished the short-term follow-up surveys (spanning from 2011 to 2013) and 1879 participants finished the long-term follow-up surveys (spanning from 2011 to 2015).

### Frailty assessment

Frailty assessment was conducted based on the widely accepted criteria initially proposed by Fried et al. (35), which were subsequently refined based on the data available in CHARLS. The definition of frailty comprised five essential elements, including exhaustion, slowness, weight loss, weakness, and low physical activity. The evaluation and definition of the five components of frailty were conducted in our study as follows: (1) Slowness: Participants were asked to self-report whether they had difficulty while walking 100-meter or climbing multiple flights of stairs without taking breaks, following a methodology similar to previous studies (30). Individuals who faced difficulty in performing climbing or walking were classified as slowness, (2) Weakness: it was assessed through self-report question “having difficulty in carrying or lifting weights exceeding 10 jin, such as heavy grocery bags” (36), (3) Exhaustion: it was considered present if the individuals responded with either “Most or all of the time” or “Occasionally or a moderate amount of the time” to anyone questions of the Center for Epidemiologic Studies-Depression scale in the Chinese version (CES-D) (37): “I could not get going during last week” or “I felt everything I did was an effort during last week.” The construction of this component was identical to that initially suggested by Fried et al. (35), (4) Weight loss: it was characterized as the inadvertent reduction of ≥5 kilograms within the past year (36), or a present body mass index (BMI) ≤ 18.5 kg/m^2^ (38), and (5) Low physical activity: WALK was defined as walking by CHARLS, referred to recreational, sporting, exercise or leisure purposes within workplaces and residences, pedestrian travel between different locations, and other forms of walking. Low physical activity represented the absence of any physical activity or WALK ≥10 min at a time in a typical week. Although differing from the component proposed by Fried et al. (35), similar treatment variables have been previously utilized to assess low physical activity (39). In our study, frailty was defined as the existence of three or more components.

### Activities of daily living

In CHARLS, the ADL scale was employed to assess the level of disability among older people. The assessment of ADL was categorized into BADL and IADL. BADL disability was described as difficulty in eating, bathing, dressing, indoor moving, toileting and continence control. IADL disability was described as difficulty in performing housework, shopping, cooking, taking medicine, and financial management. Each question is categorized into 4 possible responses: “I cannot do it,” “Yes, I have difficulty and need help,” “I have difficulty but still can do it” and “No, I do not have any difficulty” (40). The individuals were classified as experiencing ADL disability if they had inability or difficulty to complete any of the 11 items, BADL disability and IADL disability represented inability or difficulty to complete any of the items about BADL and IADL, respectively (28).

### Body measurement

The body mass index (BMI) is calculated by dividing weight (kilograms) by the square of height (meters). BMI is easy to measure and widely recorded in research, clinical nutrition, and epidemiology. We used the cut-off points standard of BMI for Chinese adults (41). BMI can be classified into 4 categories: BMI < 18.5 kg/m^2^ for underweight, BMI 18.5-24 kg/m^2^ for normal weight, BMI 24-28 kg/m^2^ for overweight and BMI ≥ 28 kg/m^2^ for obese (42–44).

### Covariates

Age/sex (female and male)/marital status/educational level/current residence /alcohol drinking/smoking status/taking activities/ physical exercise/BMI data/chronic diseases at baseline were included as covariates in our study. (1) The age groups were categorized as four groups: 45–54, 55–64, 65–74, and ≥ 75 years old, (2) Marital status was classified into married or single (including never married, divorced, separated, or widowed), (3) Educational levels range from illiterate (no formal education), below elementary school (incomplete primary education but capable of writing or reading, to graduates of home school/sishu, middle or elementary school), high school, and above vocational school (holding a two- or three-year associate/college degree, as well as a postgraduate or doctoral degree/Ph. D), (4) Current residence encompassing urban and rural, (5) Alcohol drinking encompassing more than once a month, less than once a month, and never drinker, (6) Smoking status encompassing never smoked, former-smoker and current smoker, (7) Taking activities (such as socializing with friends, providing unpaid assistance to non-cohabiting family members, neighbors or friends, participating in social/sports/other kinds of club, playing cards/chess/mahjong/participating in community club, attending the organization of community-related, engaging in charity or voluntary work, caregiving for an uncohabiting disabled or sick people without receiving compensation, attending a training or educational course, using the Internet, stocking investment) were classified into two categories: ever (at least once a month) and never, (8) Physical exercise encompassing regular physical exercises, less than regular physical exercises, and no physical exercise, and (9) Chronic diseases, including 1) hypertension, 2) high blood or sugar diabetes, 3) dyslipidemia, 4) stroke, 5) chronic lung diseases, 6) malignant tumor or cancer (excluding minor skin cancers), 7) kidney disease(except for cancer or tumor), 8) liver disease (except cancer, tumors, and fatty liver), 9) stomach or other digestive diseases (except for cancer or tumor), 10) angina, congestive heart failure, heart attack, coronary heart disease, or other heart problems, 11) nervous, emotional, or psychiatric problems, 12) asthma of self-reported (diagnosed by a doctor), 13) memory related disease, and 14) arthritis or rheumatism. By our previous criteria (45, 46), a continuous variable ranging from 0 to 14 was employed to quantify the chronic health issues in 14 common diseases. The numbers representing the chronic disease condition were categorized into 3 groups: 0, 1–2, and 3–14. These categories have been extensively employed in our prior research (39, 43–45, 47–50).

### Statistical analysis

The statistical analyses were conducted using IBM SPSS version 23.0. Categorical variables were compared using the χ^2^ test and presented as frequencies and percentages. Logistic regression was employed to analyze the odds ratios (ORs) and 95% confidence intervals (CIs) to examine the cross-sectional relationships of ADL disability and frailty in the individuals at baseline. The binary dependent variable of frailty (no-frailty and frailty) and ADL disability (no and yes) were subjected to analysis, with the regression models sequentially incorporating covariates, which were also used in our previous studies (50–52). Model 1 comprised activities of daily living disability and frailty, while model 2 additionally incorporated characteristics of socio-demographic (age, sex, marital status, educational level, current residence). Model 3 additionally encompassed health conditions and behaviors (alcohol drinking, smoking status, taking activities, physical exercise, chronic diseases). Lastly, model 4 incorporated BMI as an additional covariate. The Cox proportional hazards analysis was performed using hazard ratios (HRs) and 95% CIs to examine the prospective associations between baseline ADL disability in individuals without frailty at baseline, as well as the prospective associations between baseline frailty in individuals without ADL disability at baseline. The covariates were modeled through the same methods as those employed in cross-sectional analyses. The statistical significance of the results was determined based on a *p*-value<5%. The VIF (Variance Inflation Factor) multicollinearity diagnostic test in linear regression was conducted to detect collinearity among independent variables (53), and no collinear relationships were found between frailty and ADL (VIF < 5).

## Results

Table 1 presents the baseline characteristics of individuals according to frailty status. The mean age of participants was 61.21 (SD ± 10.19), 37.74% were male; 2.62% were above vocational school, 3.88% were in high school, and 58.27% were less than elementary school; 83.88% were single; 92.02% were living in rural; 19.35% were drinking more than once a month, and 6.96% were drinking less than once a month. 48.99% were taking activities; 24.33% were current smoking, and 8.97% were former smoking; 45.12% were regular physical exercises, and 42.80% were less than regular physical exercises; 27.48% had 3–14 chronic diseases, and 51.24% had 1–2 chronic diseases; 6.16% had BADL disability, 17.71% had IADL disability, and 19.84% had ADL disability. The frequency of frailty was 11.78%. The distribution of age, age groups, sex, educational level, marital status, alcohol drinking, taking activities, physical exercise, chronic diseases, BADL, IADL, and ADL exhibited significant differences between individuals with or without frailty.

**Table 1:** Baseline characteristics of participants according to the status of frailty in CHARLS Waves 2011 (*N*, %).

Variables	No- frailty (2321)	Frailty (310)	All participants (2631)	*t/χ2*	*p*-value
Age (years)	60.57 ± 9.93	66.00 ± 10.82	61.21 ± 10.19	−8.380	<0.001
Age groups (years)					
45–54	687 (29.60)	40 (12.90)	727 (27.63)	79.245	<0.001
55–64	865 (37.27)	105 (33.87)	970 (36.87)		
65–74	540 (23.27)	91 (29.35)	631 (23.98)		
≥75	229 (9.87)	74 (23.87)	303 (11.52)		
Sex					
Male	905 (38.99)	88 (28.39)	993 (37.74)	13.089	<0.001
Female	1,416 (61.01)	222 (71.61)	1,638 (62.26)		
Educational level					
Illiterate	770 (33.18)	157 (50.65)	927 (35.23)	39.193	<0.001
Less than elementary school	1,389 (59.84)	144 (46.45)	1,533 (58.27)		
High school	96 (4.14)	6 (1.94)	102 (3.88)		
Above vocational school	66 (2.84)	3 (0.97)	69 (2.62)		
Marital status					
Single	1963 (84.58)	244 (78.71)	2,207 (83.88)	6.961	0.008
Married	358 (15.42)	66 (21.29)	424 (16.12)		
Current residence					
Rural	2,128 (91.68)	293 (94.52)	2,421 (92.02)	2.985	0.084
Urban	193 (8.32)	17 (5.48)	210 (7.98)		
Alcohol drinking					
No	1,686 (72.64)	253 (81.61)	1939 (73.70)	12.458	0.002
Less than once a month	164 (7.07)	19 (6.13)	183 (6.96)		
More than once a month	471 (20.29)	38 (12.26)	509 (19.35)		
Smoking status					
No	1,534 (66.09)	221 (71.29)	1755 (66.70)	5.351	0.069
Former smoke	206 (8.88)	30 (9.68)	236 (8.97)		
Current smoke	581 (25.03)	59 (19.03)	640 (24.33)		
Taking activities					
No	1,155 (49.76)	187 (60.32)	1,342 (51.01)	12.202	<0.001
Yes	1,166 (50.24)	123 (39.68)	1,289 (48.99)		
Physical exercise					
No physical exercise	191 (8.23)	127 (40.97)	318 (12.09)	280.613	<0.001
Less than regular physical exercise	1,020 (43.95)	106 (34.19)	1,126 (42.80)		
Regular physical exercise	1,110 (47.82)	77 (24.84)	1,187 (45.12)		
Chronic diseases (counts)	1.70 ± 1.44	2.37 ± 1.76	1.78 ± 1.50	−6.502	<0.001
Chronic diseases groups (counts)					
0	520 (22.40)	40 (12.90)	560 (21.28)	42.331	<0.001
1–2	1,209 (52.09)	139 (44.84)	1,348 (51.24)		
3–14	592 (25.51)	131 (42.26)	723 (27.48)		
BMI (kg/m^2^)	23.75 ± 4.02	23.25 ± 4.49	23.69 ± 4.08	1.850	0.065
BADL disability					
No	2,238 (96.42)	231 (74.52)	2,469 (93.84)	227.154	<0.001
Yes	83 (3.58)	79(25.48)	162 (6.16)		
IADL disability					
No	2027 (87.33)	138 (44.52)	2,165 (82.29)	343.989	<0.001
Yes	294 (12.67)	172 (55.48)	466 (17.71)		
ADL disability					
No	1980 (85.31)	129 (41.61)	2,109 (80.16)	328.305	<0.001
Yes	341 (14.69)	181 (58.39)	522 (19.84)		

Table 2 demonstrates the baseline characteristics of individuals according to the status of BADL, IADL and ADL. A total of 2,638 BADL disability individuals 162 (6.14%) and BADL normal 2,476 (93.86%) at baseline were incorporated into the cross-sectional analysis. The distribution of age groups, educational level, smoking status, taking activities, physical exercise, and chronic diseases exhibited variations among the components of BADL. A total of 2,671 IADL disability individuals 466 (17.45%) and IADL normal 2,205 (82.55%) at baseline were incorporated into the cross-sectional analyses. The distribution of age groups, sex, educational level, current residence, alcohol drinking status, taking activities, physical exercise, chronic diseases, and BMI exhibited significant variations among the components of IADL. A total of 2,631 ADL disability individuals 522 (19.84%) and ADL normal 2,109 (80.16%) at baseline were incorporated into the cross-sectional analysis. The distribution of age groups, educational level, current residence, smoking status, taking activities, physical exercise, chronic diseases, and BMI exhibited significant differences among components of ADL.

**Table 2:** Baseline characteristics of participants according to the level of BADL, IADL and ADL in CHARLS Waves 2011 (*N*, %).

Variables	BADL disability (162)	IADL disability (466)	ADL disability (522)
Age groups(years)			
45–54	26 (16.05)	89 (19.10)	101 (19.35)
55–64	50 (30.86)	146 (31.33)	164 (31.42)
65–74	44 (27.16)	145 (31.12)	158 (30.27)
≥75	42 (25.93)	86 (18.45)	99 (18.97)
*χ2* (*p*)	42.057 (<0.001)	58.091 (<0.001)	63.518 (<0.001)
Sex			
Male	71 (43.83)	154 (33.05)	180 (34.48)
Female	91 (56.17)	312 (66.95)	342 (65.52)
*χ2* (*p*)	2.742 (0.098)	5.206 (0.023)	2.944 (0.086)
Educational level			
Illiterate	75 (46.30)	233 (50.00)	256 (49.04)
Less than elementary school	83 (51.23)	220 (47.21)	251 (48.08)
High school	2 (1.23)	9 (1.93)	10 (1.92)
Above vocational school	2 (1.23)	4 (0.86)	5 (0.96)
*χ2* (*p*)	11.821 (0.008)	59.048 (<0.001)	60.170 (<0.001)
Marital status			
Single	135 (83.33)	379 (81.33)	430 (82.38)
Married	27 (16.67)	87 (18.67)	92 (17.62)
*χ2* (*p*)	0.045 (0.832)	3.025 (0.082)	1.097 (0.295)
Current residence			
Rural	151 (93.21)	440 (94.42)	494 (94.64)
Urban	11 (6.79)	26 (5.58)	28 (5.36)
*χ2* (*p*)	0.323 (0.570)	4.532 (0.033)	6.076 (0.014)
Alcohol drinking			
No	118 (72.84)	363 (77.90)	399 (76.44)
Less than once a month	13 (8.02)	32 (6.87)	36 (6.90)
More than once a month	31 (19.14)	71 (15.24)	87 (16.67)
*χ2* (*p*)	0.293 (0.864)	6.396 (0.041)	3.083 (0.214)
Smoking status			
No	95 (58.64)	301 (64.59)	333 (63.79)
Former smoke	26 (16.05)	54 (11.59)	64 (12.26)
Current smoke	41 (25.31)	111 (23.82)	125 (23.95)
*χ2* (*p*)	11.488 (0.003)	4.991 (0.082)	8.727 (0.013)
Taking activities			
No	96 (59.26)	280 (60.09)	309 (59.20)
Yes	66 (40.74)	186 (39.91)	213 (40.80)
*χ2* (*p*)	4.685 (0.030)	18.679 (<0.001)	17.471 (<0.001)
Physical exercise			
No physical exercise	46 (28.40)	108 (23.18)	115 (22.03)
Less than regular physical exercise	59 (36.42)	177 (37.98)	203 (38.89)
Regular physical exercise	57 (35.19)	181 (38.84)	204 (39.08)
*χ2* (*p*)	43.344 (<0.001)	65.893 (<0.001)	60.858 (<0.001)
Chronic diseases(counts)			
*t* (*p*)	−6.117 (<0.001)	−8.450 (<0.001)	−9.145 (<0.001)
Chronic diseases categories			
0	11 (6.79)	56 (12.02)	60 (11.49)
1–2	80 (49.38)	218 (46.78)	247 (47.32)
3–14	71 (43.83)	192 (41.20)	215 (41.19)
*χ2* (*p*)	34.008 (<0.001)	66.055 (<0.001)	75.804 (<0.001)
BMI (kg/m^2^)			
<18.5	17 (10.49)	55 (11.80)	59 (11.30)
18.5–24	75 (46.30)	228 (48.93)	251 (48.08)
24–28	44 (27.16)	134 (28.76)	151 (28.93)
≥28	26 (16.05)	49 (10.52)	61 (11.69)
*χ2* (*p*)	3.814 (0.282)	16.285 (0.001)	12.951 (0.005)

Table 3 demonstrates baseline characteristics categorized based on the subsequent onset of frailty in short-term period (2011–2013) and long-term period (2011–2015) in BADL, IADL, and ADL cohorts. In short-term period, the likelihood of developing frailty was higher among individuals aged 65–74, residing in rural areas, having chronic diseases, and with a BMI ranging from 18.5 to 24 kg/m^2^. In long-term period, the likelihood of developing frailty was higher among individuals aged 65–74, educational level in illiterate, and having chronic diseases.

**Table 3:** Baseline characteristics classified according to subsequent onset of frailty.

Variables	2011 → 2013						2011 → 2015					
	BADL disability Incidence rate (*N* = 92, %)	*p1*	IADL disability Incidence rate (*N* = 92, %)	*p2*	ADL disability Incidence rate (*N* = 92, %)	*p3*	BADL disability |Incidence rate (*N* = 60, %)	*p1*	IADL disability Incidence rate (*N* = 60, %)	*p2*	ADL disability Incidence rate (*N* = 60, %)	*p3*
Age(years)		0.008		0.005		0.007		0.033		0.030		0.032
45–54	1.54		1.53		1.55		1.92		1.89		1.93	
55–64	2.72		2.69		2.73		2.88		2.84		2.89	
65–74	3.00		2.96		3.01		3.69		3.63		3.69	
≥75	1.09		1.08		1.09		1.12		1.10		1.12	
Sex		0.911		0.886		0.898		0.199		0.221		0.206
Male	3.09		3.05		3.10		2.40		2.37		2.41	
Female	5.27		5.21		5.28		7.21		7.10		7.22	
Educational level		0.001		0.001		0.001		0.018		0.022		0.019
Illiterate	4.09		4.04		4.10		4.97		4.89		4.98	
Less than elementary school	4.27		4.22		4.28		4.17		4.10		4.17	
High school	0.00		0.00		0.00		0.16		0.16		0.16	
Above vocational school	0.00		0.00		0.00		0.32		0.32		0.32	
Marital status		0.175		0.158		0.179		0.372		0.343		0.375
Single	6.81		6.73		6.83		8.01		7.89		8.03	
Married	1.54		1.53		1.55		1.60		1.58		1.61	
Current residence		0.021		0.021		0.021		0.100		0.105		0.099
Rural	8.17		8.08		8.20		9.46		9.31		9.47	
Urban	0.18		0.18		0.18		0.16		0.16		0.16	
Alcohol drinking		0.137		0.148		0.142		0.146		0.157		0.149
No	6.90		6.82		6.92		8.01		7.89		8.03	
Less than once a month	0.45		0.45		0.46		0.16		0.16		0.16	
More than once a month	1.00		0.99		1.00		1.44		1.42		1.44	
Smoking status		0.631		0.641		0.626		0.550		0.596		0.560
No	5.54		5.48		5.56		7.37		7.26		7.38	
Former smoke	0.54		0.54		0.55		0.48		0.47		0.48	
Current smoke	2.27		2.24		2.28		1.76		1.74		1.77	
Taking activities		0.389		0.374		0.384		0.070		0.078		0.073
No	4.54		4.49		4.55		6.09		5.99		6.10	
Yes	3.81		3.77		3.83		3.53		3.47		3.53	
Physical exercise		0.190		0.202		0.188		0.452		0.469		0.454
No physical exercise	0.64		0.63		0.64		0.64		0.63		0.64	
Less than regular physical exercise	4.45		4.40		4.46		4.97		4.89		4.98	
Regular physical exercise	3.27		3.23		3.28		4.01		3.94		4.01	
Chronic diseases(counts)		0.005		0.004		0.006		0.016		0.013		0.016
0	1.09		1.08		1.09		1.12		1.10		1.12	
1–2	3.63		3.59		3.64		4.01		3.94		4.01	
3–14	3.63		3.59		3.64		4.49		4.42		4.49	
BMI (kg/m^2^)		0.018		0.013		0.016		0.114		0.109		0.102
<18.5	1.36		1.35		1.37		1.28		1.26		1.28	
18.5–24	3.36		3.32		3.37		4.81		4.73		4.82	
24–28	2.63		2.60		2.64		2.08		2.05		2.09	
≥28	1.00		0.99		1.00		1.44		1.42		1.44	

Table 4 reports baseline characteristics classified according to the subsequent onset of BADL, IADL, and ADL disability in short-term period (2011–2013) and long-term period (2011–2015). In short-term period, the likelihood of developing BADL disability was higher among individuals aged 65–74, male, single, never smoking, having less than regular physical exercises, and having 1–2 chronic diseases. The likelihood of developing IADL disability was higher among individuals single, aged 55–64, educational level less than elementary school, having regular physical exercises, and having 1–2 chronic diseases. They tended to take no activities. The likelihood of developing ADL disability was higher among individuals aged 55–64, female, single, educational level less than elementary school, having regular physical exercises, and having 1–2 chronic diseases. They tended to never smoke and take no activities. In long-term period, the likelihood of developing BADL disability was higher among individuals aged 65–74, educational level less than elementary school, having less than regular physical exercises, and having 1–2 chronic diseases. The likelihood of developing IADL disability was higher among individuals aged 55–64, educational level less than elementary school, residing in rural areas, having less than regular physical exercises, having 1–2 chronic diseases, and with a BMI ranging from 18.5 to 24 kg/m^2^. The likelihood of developing ADL disability was higher among individuals aged 55–64, educational level with illiterate or above vocational school, residing in rural areas, having less than regular physical exercises, having 1–2 chronic diseases, and BMI ranging from 18.5 to 24 kg/m^2^.

**Table 4:** Baseline characteristics classified according to subsequent onset of BADL, IADL, and ADL disability.

Variables	2011 → 2013						2011 → 2015					
	Frailty→ BADL disability Incidence rate (*N* = 123,%)	*p1*	Frailty→ IADL disability Incidence rate (*N* = 290,%)	*p2*	Frailty→ ADL disability Incidence rate (*N* = 307,%)	*p3*	Frailty→ BADL disability Incidence rate (*N* = 143,%)	*p1*	Frailty→ IADL disability Incidence rate (*N* = 331,%)	*p2*	Frailty→ ADL disability Incidence rate (*N* = 348,%)	*p3*
Age(years)		<0.001		<0.001		<0.001		<0.001		<0.001		<0.001
45–54	1.00		2.32		3.00		0.88		3.83		4.69	
55–64	1.62		5.17		6.75		2.09		5.64		7.42	
65–74	2.41		4.49		5.87		3.14		5.53		7.16	
≥75	1.41		3.00		3.56		1.76		2.61		3.39	
Sex		<0.001		0.380		0.033		0.885		0.337		0.733
Male	3.46		6.10		8.00		2.70		6.17		7.75	
Female	2.99		8.88		11.18		5.17		11.44		14.91	
Educational level		0.559		<0.001		<0.001		0.023		<0.001		<0.001
Illiterate	2.51		6.71		8.43		3.69		8.04		11.39	
Less than elementary school	3.61		7.85		10.18		4.02		8.94		0.52	
High school	0.26		0.31		0.44		0.17		0.48		0.20	
Above vocational school	0.05		0.10		0.12		0.00		0.16		11.39	
Marital status		0.003		0.027		<0.001		0.272		0.163		0.295
Single	4.87		12.19		15.05		6.38		14.80		18.88	
Married	1.57		2.79		4.12		1.49		2.82		3.78	
Current residence		0.994		0.182		0.240		0.751		0.001		0.012
Rural	5.97		14.10		17.99		7.26		17.03		21.68	
Urban	0.47		0.88		1.19		0.61		0.59		0.98	
Alcohol drinking		0.558		0.245		0.677		0.746		0.776		0.929
No	4.56		11.52		14.62		5.89		13.20		17.12	
Less than once a month	0.47		0.88		1.06		0.66		1.06		1.50	
More than once a month	1.41		2.58		3.50		1.32		3.35		4.04	
Smoking status		<0.001		0.093		0.017		0.525		0.463		0.298
No	3.14		9.87		12.37		5.17		11.92		15.43	
Former smoke	1.00		1.70		2.31		0.61		1.60		2.08	
Current smoke	2.30		3.41		4.50		2.09		4.10		5.14	
Taking activities		0.978		<0.001		<0.001		0.120		0.133		0.187
No	3.25		8.88		11.06		4.51		9.31		11.91	
Yes	3.20		6.10		8.12		3.36		8.30		10.74	
Physical exercise		0.002		0.010		0.002		<0.001		0.047		0.033
No physical exercise	1.31		2.07		2.81		1.65		2.08		2.67	
Less than regular physical exercise	2.67		6.35		8.12		3.58		8.09		10.55	
Regular physical exercise	2.46		6.56		8.24		2.64		7.45		9.44	
Chronic diseases(counts)		0.001		<0.001		0.001		0.006		0.001		0.030
0	0.84		2.48		3.12		1.27		3.57		4.56	
1–2	2.83		7.49		9.56		3.52		8.41		11.07	
3–14	2.78		5.01		6.50		3.08		5.64		7.03	
BMI (kg/m^2^)		0.226		0.106		0.138		0.061		0.001		0.019
<18.5	0.63		1.39		1.81		0.88		1.81		2.15	
18.5–24	2.51		7.64		9.43		3.36		8.83		11.46	
24–28	2.36		4.29		5.68		2.20		4.52		5.92	
≥28	0.94		1.65		2.25		1.43		2.45		3.13	

Table 5 presents the cross-sectional relationship between ADL in baseline and frailty, frailty as the dependent variable. Both BADL (OR = 6.660, 95% CI 4.519–9.815), IADL (OR = 5.950, 95% CI 4.490–7.886), and ADL (OR = 5.658, 95% CI 4.278–7.483) disability were associated with frailty significantly after controlling for covariates of sex, age, educational level, marital status, current residence, alcohol drinking, smoking status, taking activities, physical exercise, chronic diseases, and BMI (adjusted model 4).

**Table 5:** Odds ratios (ORs) and 95% confidence interval (CIs) for frailty at baseline associated with BADL, IADL, and ADL disability at baseline.

*N* = 2,631	Model 1 OR (95% CI)	Wald, df	*p*-value	Model 2 OR (95% CI)	Wald, df	*p*-value	Model 3 OR (95% CI)	Wald, df	*p*-value	Model 4 OR (95% CI)	Wald, df	*p*-value
BADL disability												
No (2469)	Ref (1.000)			Ref (1.000)			Ref (1.000)			Ref (1.000)		
Yes (162)	9.221 (6.586, 12.911)	167.395, 1	<0.001	8.288 (5.817, 11.808)	137.070, 1	<0.001	6.588 (4.478, 9.693)	91.563, 1	<0.001	6.660 (4.519, 9.815)	91.815, 1	<0.001
IADL disability												
No (2165)	Ref (1.000)			Ref (1.000)			Ref (1.000)			Ref (1.000)		
Yes (466)	8.593 (6.658, 11.092)	272.880, 1	<0.001	7.231 (5.560, 9.404)	217.697, 1	<0.001	6.025 (4.547, 7.984)	156.399, 1	<0.001	5.950 (4.490, 7.886)	153.997, 1	<0.001
ADL disability												
No (2109)	Ref (1.000)			Ref (1.000)			Ref (1.000)			Ref (1.000)		
Yes (522)	8.147 (6.323, 10.497)	263.255, 1	<0.001	6.860 (5.283, 8.907)	208.913, 1	<0.001	5.714 (4.322, 7.556)	149.546, 1	<0.001	5.658 (4.278, 7.483)	147.638, 1	<0.001

Model 1: unadjusted.

Model 2: adjusted for age, sex, educational level, marital status, current residence.

Model 3: adjusted for age, sex, educational level, marital status, current residence, alcohol drinking, smoking status, taking activities, physical exercise, chronic diseases.

Model 4: adjusted for age, sex, educational level, marital status, current residence, alcohol drinking, smoking status, taking activities, physical exercise, chronic diseases, BMI.

Table 6 presents the cross-sectional relationship between baseline frailty and ADL, ADL as the dependent variable. Frailty was significantly associated with BADL (OR = 6.741, 95% CI 4.574–9.933), IADL (OR = 6.042, 95% CI 4.555–8.016) and ADL (OR = 5.735, 95% CI 4.333–7.591) disability after controlling for covariates of sex, age, marital status, educational level, current residence, alcohol drinking, smoking status, taking activities, physical exercise, chronic diseases, and BMI (adjusted model 4).

**Table 6:** Odds ratios (ORs) and 95% confidence interval (CIs) for BADL, IADL, and ADL disability at baseline associated with frailty at baseline.

	*N* = 2,631	Model 1 OR (95% CI)	Wald, df	*p*-value	Model 2 OR (95% CI)	Wald, df	*p*-value	Model 3 OR (95% CI)	Wald, df	*p*-value	Model 4 OR (95% CI)	Wald, df	*p*-value
Frailty→BADL disability	No (2321)	Ref (1.000)			Ref (1.000)			Ref (1.000)			Ref (1.000)		
	Yes (310)	9.221 (6.586, 12.911)	167.395, 1	<0.001	8.367 (5.870, 11.925)	138.0391	<0.001	6.676 (4.536, 9.826)	92.712, 1	<0.001	6.741 (4.574, 9.933)	93.042, 1	<0.001
Frailty→IADL disability	No (2321)	Ref (1.000)			Ref (1.000)			Ref (1.000)			Ref (1.000)		
	Yes (310)	8.593 (6.658, 11.092)	272.880, 1	<0.001	7.248 (5.572, 9.429)	217.861, 1	<0.001	6.114 (4.609, 8.110)	157.720, 1	<0.001	6.042 (4.555, 8.016)	155.607, 1	<0.001
Frailty→ADL disability	No (2321)	Ref (1.000)			Ref (1.000)			Ref (1.000)			Ref (1.000)		
	Yes (310)	8.147 (6.323,10.497)	263.255, 1	<0.001	6.878 (5.296, 8.932)	209.096, 1	<0.001	5.789 (4.374, 7.663)	150.683, 1	<0.001	5.735 (4.333, 7.591)	149.099, 1	<0.001

Model 1: unadjusted.

Model 2: adjusted for age, sex, educational level, marital status, current residence.

Model 3: adjusted for age, sex, educational level, marital status, current residence, alcohol drinking, smoking status, taking activities, physical exercise, chronic diseases.

Model 4: adjusted for age, sex, educational level, marital status, current residence, alcohol drinking, smoking status, taking activities, physical exercise, chronic diseases, BMI.

Table 7 reports the prospective associations between baseline ADL and frailty after 2 years and 4 years of follow-up in the individuals without frailty at baseline. First, in crude analysis, IADL and ADL disability showed a significant association with incident frailty during the short-term [IADL disability: HR = 2.603 (1.563, 4.335); ADL disability: HR = 2.499 (1.533, 4.072)]. After controlling for covariates of age, sex, marital status, educational level, current residence, alcohol drinking, smoking status, taking activities, physical exercise, chronic diseases, and BMI, IADL disability [HR = 1.971 (1.150, 3.379)] and ADL disability [HR = 1.920 (1.146, 3.215)] showed a significant association with incident frailty during the short-term. Second, in crude analysis, IADL and ADL disability showed a significant association with incident frailty during the long-term [IADL disability: HR = 2.685 (1.472, 4.898); ADL disability: HR = 2.111 (1.165, 3.826)]. After controlling for covariates of age, sex, educational level, marital status, and current residence, IADL and ADL disability showed significant association with incident frailty [IADL disability: HR = 2.498 (1.349, 4.626); ADL disability: HR = 1.873 (1.018, 3.448)] (adjusted model 2). After adjusting for all covariates, IADL disability [HR = 2.056 (1.085, 3.895)] still showed a significant association with incident frailty, but the ADL disability [HR = 1.556 (0.830, 2.917)] did not show significant association with incident frailty (adjusted model 4).

**Table 7:** Association between BADL, IADL, and ADL disability and incident frailty without frailty at baseline.

Follow-up period			Model 1 HR (95% CI)	Wald, df	*p*-value	Model 2 HR (95%CI)	Wald, df	*p*-value	Model 3 HR (95%CI)	Wald, df	*p*-value	Model 4 HR (95%CI)	Wald, df	*p*-value
2011 → 2013	*N* = 1,101	BADL disability												
		No (1060)	Ref (1.000)			Ref (1.000)			Ref (1.000)			Ref (1.000)		
		Yes (41)	1.942 (0.794, 4.745)	2.117, 1	0.146	1.865 (0.750, 4.639)	1.799, 1	0.180	1.625 (0.641, 4.116)	1.048, 1	0.306	1.632 (0.644, 4.134)	1.065, 1	0.302
	*N* = 1,114	IADL disability												
		No (975)	Ref (1.000)			Ref (1.000)			Ref (1.000)			Ref (1.000)		
		Yes (139)	2.603 (1.563, 4.335)	13.524, 1	<0.001	2.246 (1.333, 3.786)	9.236, 1	0.002	1.985 (1.159, 3.398)	6.245, 1	0.012	1.971 (1.150, 3.379)	6.094, 1	0.014
	*N* = 1,098	ADL disability												
		No (935)	Ref (1.000)			Ref (1.000)			Ref (1.000)			Ref (1.000)		
		Yes (163)	2.499 (1.533, 4.072)	13.514, 1	<0.001	2.134 (1.295, 3.517)	8.852, 1	0.003	1.930 (1.153, 3.230)	6.256, 1	0.012	1.920 (1.146, 3.215)	6.149, 1	0.013
2011 → 2015	*N* = 624	BADL disability												
		No (600)	Ref (1.000)			Ref (1.000)			Ref (1.000)			Ref (1.000)		
		Yes (24)	0.399 (0.053, 3.006)	0.796, 1	0.372	0.346 (0.045, 2.628)	1.054, 1	0.305	0.315 (0.041, 2.411)	1.238, 1	0.266	0.318 (0.042, 2.435)	1.216, 1	0.270
	*N* = 634	IADL disability												
		No (537)	Ref (1.000)			Ref (1.000)			Ref (1.000)			Ref (1.000)		
		Yes (97)	2.685 (1.472, 4.898)	10.376, 1	0.001	2.498 (1.349, 4.626)	8.473, 1	0.004	2.094 (1.109, 3.956)	5.187, 1	0.023	2.056 (1.085, 3.895)	4.888, 1	0.027
	*N* = 623	ADL disability												
		No (510)	Ref (1.000)			Ref (1.000)			Ref (1.000)			Ref (1.000)		
		Yes (113)	2.111 (1.165, 3.826)	6.068, 1	0.014	1.873 (1.018, 3.448)	4.068, 1	0.044	1.583 (0.845, 2.963)	2.059, 1	0.151	1.556 (0.830, 2.917)	1.899, 1	0.168

Model 1: unadjusted.

Model 2: adjusted for age, sex, educational level, marital status, current residence.

Model 3: adjusted for age, sex, educational level, marital status, current residence, alcohol drinking, smoking status, taking activities, physical exercise, chronic diseases.

Model 4: adjusted for age, sex, educational level, marital status, current residence, alcohol drinking, smoking status, taking activities, physical exercise, chronic diseases, BMI.

Table 8 reports the prospective associations between frailty in baseline and ADL after 2 years and 4 years of follow-up in individuals without ADL disability in baseline. First, in crude analysis, frailty showed a significant association with incident BADL disability [frailty: HR = 2.044 (1.256, 3.327)], IADL disability [frailty: HR = 2.069 (1.348, 3.177)] and ADL disability [frailty: HR = 1.865(1.209, 2.876)] during the short-term. After controlling for covariates of age, sex, marital status, educational level, and current residence, frailty was significantly associated with incident BADL disability [frailty: HR = 2.008 (1.210, 3.334)], IADL disability [frailty: HR = 1.740 (1.110, 2.725)], and ADL disability [frailty: HR = 1.703 (1.085, 2.671)] (adjusted model 2). After adjusting for all covariates, frailty was not significantly associated with incident BADL disability [frailty: HR = 1.573 (0.912, 2.712)], IADL disability [frailty: HR = 1.437 (0.901, 2.291)], and ADL disability [frailty: HR = 1.393 (0.873, 2.225)] during the short-term (adjusted model 4). Second, in crude analysis, frailty was significantly associated with incident BADL disability [frailty: HR = 3.042 (1.982, 4.669)], IADL disability [frailty: HR = 2.364 (1.560, 3.582)] and ADL disability [frailty: HR = 1.840 (1.187, 2.852)] during the long-term. After adjusting for all covariates, frailty was significantly associated with incident BADL disability [frailty: HR = 1.820 (1.126, 2.939)] and IADL disability [frailty: HR = 1.724 (1.103, 2.694)]. However, frailty did not show a significant association with incident ADL disability [frailty: HR = 1.351 (0.846, 2.158)] during the long-term (adjusted model 4).

**Table 8:** Association between frailty and incident BADL, IADL and ADL disability without disability at baseline.

Follow-up period			Model 1 HR (95% CI)	Wald, df	*p*-value	Model 2 HR (95% CI)	Wald, df	*p*-value	Model 3 HR (95% CI)	Wald, df	*p*-value	Model 4 HR (95%CI)	Wald, df	*p*-value
2011 → 2013	BADL disability	Frailty (*N* = 1909)												
		No (1715)	Ref (1.000)			Ref (1.000)			Ref (1.000)			Ref (1.000)		
		Yes (194)	2.044 (1.256, 3.327)	8.271, 1	0.004	2.008 (1.210, 3.334)	7.268, 1	0.007	1.507 (0.877, 2.590)	2.202, 1	0.138	1.573 (0.912, 2.712)	2.658, 1	0.103
	IADL disability	Frailty (*N* = 1936)												
		No (1815)	Ref (1.000)			Ref (1.000)			Ref (1.000)			Ref (1.000)		
		Yes (121)	2.069 (1.348, 3.177)	11.047, 1	0.001	1.740 (1.110, 2.725)	5.844, 1	0.016	1.449 (0.909, 2.310)	2.425, 1	0.119	1.437 (0.901, 2.291)	2.318, 1	0.128
	ADL disability	Frailty (*N* = 1,601)												
		No (1493)	Ref (1.000)			Ref (1.000)			Ref (1.000)			Ref (1.000)		
		Yes (108)	1.865 (1.209, 2.876)	7.948, 1	0.005	1.703 (1.085, 2.671)	5.365, 1	0.021	1.401 (0.878, 2.235)	1.995, 1	0.158	1.393 (0.873, 2.225)	1.930, 1	0.165
2011 → 2015	BADL disability	Frailty (*N* = 1818)												
		No (1641)	Ref (1.000)			Ref (1.000)			Ref (1.000)			Ref (1.000)		
		Yes (177)	3.042 (1.982, 4.669)	25.887, 1	<0.001	2.311 (1.473, 3.624)	13.302, 1	<0.001	1.807 (1.121, 2.912)	5.902, 1	0.015	1.820(1.126, 2.939)	5.983, 1	0.014
	IADL disability	Frailty (*N* = 1879)												
		No (1767)	Ref (1.000)			Ref (1.000)			Ref (1.000)			Ref (1.000)		
		Yes (112)	2.364 (1.560, 3.582)	16.441, 1	<0.001	1.963 (1.274, 3.024)	9.348, 1	0.002	1.732 (1.108, 2.707)	5.817, 1	0.016	1.724 (1.103, 2.694)	5.715, 1	0.017
	ADL disability	Frailty (*N* = 1,536)												
		No (1439)	Ref (1.000)			Ref (1.000)			Ref (1.000)			Ref (1.000)		
		Yes (97)	1.840 (1.187, 2.852)	7.436, 1	0.006	1.544 (0.978, 2.438)	3.484, 1	0.062	1.361 (0.852, 2.173)	1.662, 1	0.197	1.351 (0.846, 2.158)	1.585, 1	0.208

Model 1: unadjusted.

Model 2: adjusted for age, sex, educational level, marital status, current residence.

Model 3: adjusted for age, sex, educational level, marital status, current residence, alcohol drinking, smoking status, taking activities, physical exercise, chronic diseases.

Model 4: adjusted for age, sex, educational level, marital status, current residence, alcohol drinking, smoking status, taking activities, physical exercise, chronic diseases, BMI.

## Discussion

The study based on a representative sample from CHARLS reveals ADL disability and frailty are common among middle-aged and older individuals in China (6). There is a positive, reciprocal, time-varying association between ADL disability and frailty. The present study provides the comprehensive analysis of the reciprocal associations between ADL/IADL/BADL and frailty, both longitudinally and cross-sectionally. First, there was a confirmed correlation between baseline ADL/IADL/BADL disability and frailty, as well as between baseline frailty and ADL/BADL/IADL disability. Second, ADL/IADL disability at baseline was reported to be associated with the onset of frailty significantly after a two-year follow-up. However, no significant associations were found between baseline frailty and the onset of ADL/BADL/IADL disability after a two-year follow-up. Last, Baseline IADL disability was significantly associated with the onset of frailty after a four-year follow-up. In contrast, the baseline frailty was significantly associated with the onset of BADL and IADL disability after a four-year follow-up.

The meta-analysis study (9) including cross-sectional and cohort studies, comprehensive analysis showed that frailty increased the likelihood of developing IADL and BADL disabilities. In terms of follow-up time, frailty was significantly associated with an elevated risk of disability onset in BADL and IADL after 2-5 years and above 5 years of follow-up, which was in line with our results after 4 years of follow-up; but no relevant data was found at 1–2 years of follow-up. Thus, further studies during short-term and long-term follow-up are needed to explore the dynamic association between frailty and incidence of ADL disability in middle-aged and older adults. Previous meta-analysis (30, 31) including studies from United States, the United Kingdom, Europe, Australia, Mexico, Korea and China revealed that during 1–15 years of follow-up, frailty individuals had a higher risk of both ADL and IADL disability compared to those without frailty among middle-aged and older people. In contrast, there was no significant association between frailty in baseline and the onset of ADL/BADL/IADL disability after a short-term (2-year) period in our study. The occurrence can be elucidated by the cumulative effect, demonstrating significant association between frailty and the longitudinal onset of BADL and IADL disability over a long-term (4-year) period.

Similarly, previous studies have also reported that disability in ADL/BADL/IADL is associated with frailty in cross-section (6, 34, 54, 55), which aligns with our results. Other research reported that BADL and IADL disability at baseline did not serve as predictors for the occurrence of frailty after one-year follow-up in community-dwelling older adults (1). This study is conducted in middle-aged and older people, IADL and ADL disability at baseline serve as predictors for the occurrence of frailty after short-term follow-up, IADL disability at baseline serves as predictors for the onset of frailty after long-term follow-up among middle-aged and older individuals. The occurrence can be ascribed to the cumulative effect.

In theory, two directions of this relationship are reasonable according to middle-aged and older individuals. Through various potential mechanisms, frailty can exert an impact on ADL disability. For instance, frailty individuals commonly had a higher prevalence of chronic diseases than non-frailty people (56). People with chronic conditions demonstrated higher incidence rates of disability across all ADL items (57–59), our results also supported this view. Over time, the decline of BADL such as bathing, walking, toileting, dressing, transferring and eating, as well as IADL such as performing housework, shopping, cooking, taking medicine and financial management will increase. IADL represent the functional competence required for complex everyday tasks, such as shopping or meal preparation, and are recognized to decline before BADL associated with self-care (60). Besides, frailty phenotype demonstrated independent predictability for ADL disability. The presence of frailty syndrome may serve as an etiologic factor and physiological precursor in the development of disability, owing to its primary characteristics encompassing weakness, exhaustion, weight loss, slowness, and low physical activity. Frailty was likely to primarily impact functions reliant on energy expenditure and speed of execution (e.g., mobility), frailty initially affected mobility tasks before manifesting difficulties in BADL and IADL (9, 35, 56). Additionally, a 2-year longitudinal study revealed that individuals with weakness faced a significantly incidence risk of ADL disability compared to those without weakness individuals at baseline in the older (61). A cohort study indicated that frailty acts as a significant negative factor in the recovery process of disability in ADL among newly disabled Chinese older people (62). Furthermore, frailty is characterized by a decline in the body’s reserve capacity, implying physiological decrements and adverse health outcomes can be anticipated (63). The underlying mechanism involves a multifaceted interaction, including a lower activity level, malnutrition, sarcopenia, weight loss, and difficulty maintaining homeostasis (35). This multiorgan system imbalance manifests as impaired energy metabolism and neuromuscular alterations (64), along with the growth of age, potentially leading to a decline in physical functioning as well as an increased occurrence of ADL (65). Simultaneously, existing research has reported that engagement in physical activities can reduce the risk of ADL disability and functional limitations among older individuals (66, 67), our findings also support this view, one of the characteristics of frailty is a decrease in physical activity, which would increase the risk of ADL disability.

Similarly, previous studies have also demonstrated that disability in ADL is a necessary predictor of frailty (6, 34). Individuals who had difficulty with ADL were more likely to show having frailty (55). Through a variety of underlying mechanisms, ADL disability can affect frailty. For example, having more difficulties with BADL and IADL increases the risk of frailty by limiting older people’s social participation and physical activity. People with higher levels of ADL functioning tend to experience more social participation (68), and individuals with ADL disabilities experience the opposite pattern. Disability in BADL, e.g., eating, bathing, dressing, and toileting, etc., as well as IADL, e.g., shopping, taking medicine and financial management, etc., can act as a hindrance that causes or exacerbates low level of social participation (69), may results social isolation and loneliness (70), accordingly promoting the frailty progression (71, 72). Low physical activity poses a significant risk factor for the development of frailty (73, 74). Difficulties in ADL can impede mobility and hinder flexibility, inevitably leading to insufficient levels of physical activity and reduced function of extremities, and therefore increasing the risk of physical frailty as people age (75, 76). People with ADL difficulties tend to experience more negative affect (68), which is a risk factor for frailty (77). Disability in ADL, such as continence control, housework and cooking, can serve as a stressor due to the loss of independence and decline in self-care abilities, causing or exacerbating high level of negative affect (69), and increasing the incidence risk of frailty (21, 72). The inability to perform essential ADL leads to dependence on others and/or mechanical aids, and negatively affects daily activities, safe conditions and quality of life (78), all those increase slowness, weakness, low physical activity, and exhaustion resulting in frailty (79, 80). In addition, the middle-aged and older participants with disability in ADL often suffer from chronic diseases (59), and the ability to take medicine in IADL may be restricted, which is not conducive to disease control, thus aggravating physical weakness and presenting frailty (3, 6).

Frailty is characterized by a condition of increased vulnerability to both exogenous and endogenous stressors, impacting the quality of life and raising the risk of adverse health-related outcomes for individuals (81, 82), and frailty is significantly associated with mortality (3). Frailty is described as a transition state between ADL disability and successful aging (83). Identifying potential frailty in middle-aged and older population is essential for early prevention and management. Special attention should be given to middle-aged and older person with ADL disability to prevent the incidence of their frailty. Besides, ADL disability is one independent risk factor for 7 years mortality in middle-aged and older adults in China (84), and ADL skills can facilitate successful aging (85). Frailty serves as a strong predictor for disability in ADL, while disability in ADL is also a strong predictor of frailty among middle-aged and older population. The findings of our research demonstrate that middle-aged and older individuals with ADL disability and frailty are both at high risk and require increased attention. The present study just examines the bivariate bidirectional association between frailty and ADL disability. Further studies are necessary to determine the impact of this relationship in order to break this vicious cycle.

### Strengths and limitations of the study

This study possesses several strengths. It is based on a nationwide representative cohort with a relatively large sample size, which includes individuals aged ≥45 years. It explores the bidirectional association between frailty and ADL disability across two different time intervals and provides help to understand the short-term and long-term effects further. Certain limitations should be noted in the current study. Some predictors in our study, such as ADL, frailty, and disease history, were self-reported and potentially introduced partial information bias. The exclusion of some participants with incomplete data resulted in a reduced sample size and would affect the analysis results. This study did not investigate how certain lifestyles might relate to ADL disabilities, which should be carried out in the follow-up study.

## Conclusion

In summary, this study established bidirectional cross-sectional and longitudinal associations between frailty and ADL using a nationwide representative sample in China. This study suggested that disability in ADL increased the risk of frailty and vice versa. They may create a vicious circle, negatively reinforcing each other. Early interventions targeting ADL disability or frailty for middle-aged and older adults will be beneficial to improving their health status. Accordingly, in the context of increasing aging, the interaction between ADL disability and frailty should be explored to prevent the development of a vicious cycle.

## Data availability statement

The datasets presented in this study can be found in online repositories. The names of the repository/repositories and accession number(s) can be found at: https://charls.charlsdata.com/pages/data/111/zh-cn.html.

## Author contributions

XiaopL: Writing – original draft, Writing – review & editing. XiaogL: Writing – review & editing. LS: Writing – review & editing. LY: Writing – review & editing. CW: Writing – review & editing. TY: Writing – review & editing. YL: Writing – review & editing. JL: Writing – review & editing. ML: Writing – review & editing. DZ: Writing – review & editing. YH: Writing – review & editing. HL: Writing – review & editing. LZ: Writing – review & editing.
